# The Effect of Strabismus Muscle Surgery on Corneal Biomechanics

**DOI:** 10.1155/2018/8072140

**Published:** 2018-09-16

**Authors:** Heba A. El Gendy, Noha M. Khalil, Iman M. Eissa, Shireen MA. Shousha

**Affiliations:** ^1^Professor of Ophthalmology, Faculty of Medicine, Cairo University, Cairo, Egypt; ^2^Associate Professor of Ophthalmology, Faculty of Medicine, Cairo University, Cairo, Egypt; ^3^Lecturer of Ophthalmology, Faculty of Medicine, Cairo University, Cairo, Egypt

## Abstract

**Purpose:**

Studying the early effect of different extraocular muscle (EOM) surgeries on corneal biomechanics.

**Subjects and methods:**

This is a prospective, nonrandomized, interventional study, in which 42 eyes of 29 candidates for EOM surgery for strabismus correction at Cairo university hospitals, aged 14–37 years, were recruited. All participants had measuring of the visual acuity, refraction (spherical equivalent (SE)), assessment of the EOM motility and muscle balance, sensory evaluation, fundus examination, and assessing the ocular biomechanics using the Ocular response analyzer (ORA, Reichert, INC., Depew, NY) noting the corneal hysteresis (CH) and corneal resistance factor (CRF) preoperatively. Same patients were reassessed using ORA 4 weeks postoperatively following a different standard EOM surgery (recti weakening/strengthening and inferior oblique weakening either (graded recession) according to the surgical indication, and ∆CH and ∆CRF were calculated, each is the preoperative − the postoperative value.

**Results:**

∆CH and ∆CRF = −0.78 ± 1.56 and −0.72 ± 2.15, respectively, and a highly significant difference was found between each of the pre- and postoperative CH and CRF (*p* < 0.001). 18 eyes had single EOM surgery, while 24 had multiple (2 or 3) EOM surgery; ∆CH in the single group = −1.28 ± 1.5, and ∆CH in the multiple group = 0.4 ± 1.49 (*p*=0.07). 23 eyes had EOM weakening surgery, while 18 had combined weakening and strengthening EOM surgery: ∆CH in the weakening group = −1.24 ± 1.77 and ∆CH in combined group = −0.26 ± 1.07 (*p*=0.04). A nonsignificant difference was found for ∆CRF (*p*=0.53).

**Conclusion:**

A different EOM surgery has an early tendency for increase of the postoperative CH specially for muscle weakening procedures (recti recession/inferior oblique muscle weakening).

## 1. Introduction

Corneal and ocular biomechanics have been a topic of increasing interest in ophthalmology over the last two decades. The eye has been commonly thought of as an optical rather than a mechanical system; however, biomechanics can still play an important role in a number of different ophthalmic pathologies [1, 2].

Corneal biomechanical properties, namely, corneal hysteresis (CH), corneal resistance factor (CRF), and corneal compensated intraocular pressure (IOPcc), generally reflect corneal deformation and equilibrium under the application of external force. Hence, the structure and properties of corneal tissue are dependent on the nature of the components present in it and their relative amounts. Mechanical properties of a tissue will thus depend on how fibers, cells, and ground substance are structurally organized into this tissue [3–5]. One commercially available and approved instrument used to measure CH, CRF, and IOPcc is the Ocular Response Analyzer (ORA, Reichert, Inc., Depew, NY) [6, 7].

In the past years, multiple studies have tackled the fact that corneal topography and corneal refractive power can change after strabismus surgery. However, some of the refractive changes were found to be mild and regress with time [8–10].

Recent studies found mild corneal topographic changes after sutureless vitrectomy as well, that most of these changes however, decayed with time [11, 12].

For the current study, the authors wanted to test a new hypothesis triggered by these previous studies as to whether corneal biomechanics can be affected by strabismus muscle surgery. To our knowledge, there is paucity of literature on this point. The authors postulate that if muscle tension forces applied to the global wall were changed, this could have a possible effect on corneal hysteresis, IOPg, IOPcc, and corneal resistance factor.

## 2. Subjects and Methods

The present study was approved by the Research Ethics Committee of the Faculty of Medicine, Cairo University. Data collection conformed to all local laws, and the study followed the guidelines of the Declaration of Helsinki 1964 [13].

In this prospective, nonrandomized, interventional study, 29 patients, aged 14–37 years, who presented with manifest heterotropias and who met the inclusion criteria for the current study, were scheduled for elective strabismus muscle surgery. All cases were recruited from the outpatient strabismus clinic in Kasr Al Ainy Hospital, Faculty of Medicine, Cairo University, during the period from December 2016 to July 2017.

All patients who met the predetermined inclusion criteria or their guardians were requested to sign a full informed consent regarding their acceptance of participation in the current study, the surgical procedure, the follow-up protocol regimen, and the possible complications.

### 2.1. Inclusion Criteria

The following are the various inclusion criteria:

- Manifest primary comitant heterotropia
- No previous history of strabismus surgery or any other ocular surgery
- No history of ocular conditions that may affect ocular biomechanics, i.e., corneal scars, keratoconus, or glaucoma
- No history of contact lens wear
- No history of systemic conditions that may affect the ocular biomechanics, i.e., diabetes mellitus, thyroid dysfunction, or collagen vascular diseases

Full history taking regarding the onset of eye deviation, its duration, history of wearing glasses, amblyopia therapy, and previous ocular surgeries was taken from all patients.

All patients underwent full ophthalmological examination, as well as full motor and sensory assessment of their heterotropias, and cases with paralytic or restrictive strabismus were excluded, as well as cases with previous muscle surgeries.

### 2.2. Preoperative Measurement of Corneal Biomechanics

Corneal hysteresis (CH), corneal resistance factor (CRF), intraocular pressure Goldmann (IOPg), and cornea compensated intraocular pressure (IOPcc) were measured using Reichert Ocular Response Analyzer (ORA, Reichert Instruments, Depew, New York, USA). Measurements were done for all patients between 10 AM and 12 PM by the same masked operator to avoid bias induced by diurnal variations in corneal biomechanics [9]. Three consecutive measurements were performed, and the best waveform score (WS) from each patient was included in the statistical analysis.

### 2.3. The Surgical Intervention

All patients underwent strabismus muscle surgery under general anesthesia in the form of either muscle weakening procedure (recession of recti or inferior oblique graded recession,) or strengthening procedure (muscle resection or plication), with the determined amount of recession and/or resection in millimeters done according to the preoperative angle measurements.

All cases were done using a low magnification power surgical microscope, through fornix-based conjunctival incisions.

Out of 42 eyes that underwent surgery, 17 eyes underwent a combined weakening-strengthening procedure, 24 eyes underwent muscle weakening procedure, and only one eye underwent a single muscle strengthening procedure.

The number of the eyes which underwent single muscle surgery was 18: 17 with muscle weakening procedure and a single eye with a single muscle strengthening procedure, while 24 eyes underwent two or three muscle surgeries: 18 eyes out of 24 underwent combined weakening-strengthening and 6 eyes underwent combined muscle weakening procedures (rectus and oblique muscles).

### 2.4. Postoperative Follow-Up

All patients were routinely examined 1^st^ day postoperatively, regarding their ocular alignment, ocular motility, and conjunctival wound coaptation.

All patients received routine medications in the form of combined topical steroids and antibiotics preparations 4 times daily for 2 weeks' duration, and all were requested to attend their follow-up visits regularly at 2 weeks and 4 weeks postoperatively.

### 2.5. Postoperative Measurement of Corneal Biomechanics

All patients underwent a second postoperative measurement of corneal biomechanics at 4 weeks postoperatively, to be compared with those recorded preoperatively, and measurements were done for all patients between 10 AM and 12 PM to avoid bias induced by diurnal variations in corneal biomechanics [9].

The measurements were recorded by the same masked operator who was concerned with the preoperative measurements.

### 2.6. Data Collection and Statistical Analysis

Statistical analysis was performed using Statistical Package for Social Sciences, version 16 (SPSS 16). All variables were tested for normality using the Kolmogorov–Smirnov test, that was nonsignificant, and normality was accepted for all variables. Accordingly, quantitative data are presented as mean ± standard deviation, while qualitative data are presented as number (percentage). Because all quantitative data were normally distributed, variables were compared between two related samples using 2-sample *t*-test, and the probability value (*p* ≤ 0.05) was considered statistically significant.

Bivariate correlations were performed between different parameters using Pearson's correlation coefficient (*r*), and Δ for a variable was calculated by subtracting the preoperative value from the postoperative value.

## 3. Results

In this prospective nonrandomized interventional study, 42 eyes of 29 patients (14 males and 15 females) aged 14–37 years (mean 24.9 ± 6.86 SD), who met the inclusion criteria of the proposed study protocol and scheduled for elective strabismus muscle surgery, were recruited for participation in the study.

Twenty patients presented with exotropia (mean 52.5 ± 1.19 Δ), 6 presented with esotropia (mean 33.75 ± 1.37 Δ), and 3 with hypertropia (mean 21.6 ± 2.9 Δ), whereas the mean preoperative spherical equivalent was −2.56 ± 3.16 D.

All the eyes underwent elective muscle surgeries through either muscle weakening (mean 8.17 ± 1.87 mm) or strengthening procedures (mean 6. 46 ± 0.82 mm and 5.41 ± 0.73 mm) for muscle resection and plication, respectively, according to the preoperative measurements of the angles of deviations.

The preoperative parameters (preoperative intraocular pressure Goldmann (IOPg), preoperative intraocular pressure corneal compensated (IOPcc), corneal hysteresis (pre-op CH), and corneal resistance factor (pre-op CRF)) were compared to the postoperative parameters (post-op IOPg, post-op IOPcc, post-op CH, and post-op CRF) of the same studied eyes, measured at the 4th postoperative week.

A statistically significant difference in CH, CRF, and IOPg was noted, as well as a tendency for a change in IOPcc as well which was close to being significant (*p*=0.06).

The comparison between preoperative and the postoperative parameters of the same studied eyes is summarized in Table 1.

The change in corneal hysteresis (CH) and in corneal resistance factor (CRF) was calculated as the preoperative-postoperative value (ΔCH and ΔCRF); both were negative values, i.e., −0.78 ± 1.56 and −0.72 ± 2.15, respectively, denoting a postoperative mean increase in CH and CRF.

Bivariate correlations between ΔCH and each of age, sex, refraction, and preoperative CH (Table 2) were all nonsignificant except for a significant fair positive linear correlation to preoperative CH.

Bivariate correlations between ΔCRF and each of age, sex, refraction, and preoperative CRF (Table 3) were all not significant.

Comparison of the differences between pre- and postoperatively studied corneal biomechanics parameters for the eyes with single muscle surgery (*n*=18), which were mostly muscle recessions—17 out 18 eyes, versus more than 2 or 3 muscle surgeries (*n*=24) is shown in Table 4 where we recorded higher postoperative mean increase in CH and CRF in the eyes with single muscle surgery compared to multiple muscle surgeries, that was found to be statistically significant regarding CH (*p* ≤ 0.07).

Furthermore, comparison of the differences between pre- and postoperatively studied corneal biomechanics parameters for the eyes with weakening muscle surgery (*n*=23, recti recession or inferior oblique muscle weakening) versus combined (weakening and strengthening) muscle surgery (*n*=18) is shown in Table 5, with the results showing a significant postoperative increase in corneal hysteresis for the eyes with muscle weakening surgery compared to those with the combined weakening-strengthening procedure (*p* ≤ 0.04), yet no statistically significant difference regarding CRF was shown in the same group of patients.

## 4. Discussion

Corneal biomechanics are relatively new parameters recently found to have clinical implications when tackling certain ocular conditions including glaucoma and some refractive surgeries. Namely, these corneal biomechanical properties are corneal hysteresis, corneal resistance factor, and corneal compensated IOP [7, 14].

In the current study, CH, CRF, and IOPg were all found to increase significantly early after strabismus surgery, a finding which supports our hypothesis that, if forces applied to the global wall by the extraocular muscles are changed, the corneal biomechanics are likely to change. There was also a tendency for an increase in IOPcc after surgery (*p*=0.06); however, it was not statistically significant. The significant increase in IOPg could be partially attributed to steroid-induced rise of IOP in three cases which went back to normal with follow-up after cessation of topical steroids.

Despite the fact that to our knowledge no recent studies have tackled the same variables, our hypothesis was inspired based on older studies which stated that, after strabismus surgery, corneal topographic changes were likely to occur [8, 9, 15]. In other studies, transient corneal topographic changes were found to occur after sutureless vitrectomy as well [11, 12]. These changes occurred despite the lack of direct tissue continuity between the cornea and the vitreous body. Similarly, we believe that changing the site of insertion of extraocular muscles and/or changing the area of contact between a muscle and the globe can potentially alter corneal biomechanics.

Moreover, pterygium was noted to affect corneal biomechanical properties [16, 17]. In a recent study by Koç et al., corneal biomechanical changes were also found to occur after pterygium surgery, where all preoperative ORA measured parameters were recorded to be decreased, yet the decrease was statistically nonsignificant [18].

Another study by Mombaerts et al. [19] stated that higher degrees of “with the rule astigmatism” were found in patients under 55 years old who suffered Graves' ophthalmopathy. These patients were examined clinically and by CT and were found to have fibrosis of the orbit as well as restrictive motility disorders. Restrictive motility disorders changes the tension force a muscle exerts on the global wall, to an extent that it may induce with the rule corneal astigmatism in this study.

Furthermore, changes in the corneal biomechanical properties were reported in the eyes with thyroid eye disease (TED), that CH values showed significant lowering in TED patients as compared to controls, that were noted to be correlated to the severity of the disease “as the severity of TED increases, CH decreases” [20]. Similarly, the authors assumed that a weakening or a strengthening muscle procedure could alter the corneal response to external pressure and thus potentially affect corneal biomechanical properties.

In 2017, Jiang et al. [21] conducted a meta-analysis about corneal biomechanical properties after penetrating keratoplasty (PK) or deep anterior lamellar keratoplasty (DALK) using the ORA; they suggested that both CH and CRF had better recovery after corneal transplantation with DALK than PK.

Comparing the effect of small incision cataract surgery and phacoemulsification on corneal biomechanical properties using ORA, significant differences between preoperative and postoperative corneal biomechanical values were found for CH, IOPcc, and IOPg. The 2.2 mm coaxial microincision cataract surgery group seemed to have a faster recovery (1 week) compared to the 3.0 mm standard coaxial phacoemulsification group (took 2 weeks to return to the preoperative values) [22].

The current study found that weakening muscle procedures had a greater effect on overall corneal biomechanics, with a statistically significant postoperative increase (*p*=0.04) when compared to procedures involving muscle strengthening. Our proposed explanation for that is that the release of forces (the yielding effect of weakening procedures) on the global wall seemed to be transmitted to the corneal tissue as well leading it to become more resilient. Further studies however are needed to validate this preliminary finding.

Our study also found that changes in corneal biomechanical properties tended to be higher with single versus multiple muscle surgeries (*p*=0.07). However, the difference was only close to being significant. The authors postulate that the unopposed mechanical effect of a single muscle surgery would naturally be more pronounced than if this effect was somehow “neutralized” by multiple muscle surgeries where the weakening of a lateral rectus, for example, is somehow neutralized by strengthening the ipsilateral medial rectus muscle.

There are limitations to the current study; the relatively small number of cases and the short follow-up period are definite limitations. A continuation of this study is planned by the authors where we can include a larger number of patients and repeat follow-up after longer time intervals. The authors intend to do more extensive subgrouping in the upcoming study, where patients undergoing different single muscle procedures can be further compared with each other and with others undergoing multiple muscle procedures and where weakening procedures can be compared to strengthening ones in more detail. A longer follow-up period will shed more light on whether these postoperative changes in corneal biomechanics are temporary and will decay with time or will become permanent postoperative findings.

We believe this study touches upon a potentially interesting finding exploring the relationship between corneal biomechanics and extraocular muscle forces, a point which can have important clinical implications in patients who underwent strabismus muscle surgery and are candidates for refractive surgery or are being evaluated as glaucoma suspects. New parameters derived from understanding the complex relationship between different ocular tissues utilizing corneal biomechanics may act as a tool for guiding better safety and efficacy of different eye healthcare procedures.

## Figures and Tables

**Table 1:** The comparison between preoperative and postoperative parameters.

Parameter (mmHg)	Preoperative	Postoperative	*p* value
IOPg	13.32 ± 3.12	13.39 ± 5.76	0.009
IOPcc	14.4 ± 3.01	14.12 ± 5	0.067
CH	9.9 ± 1.78	10.69 ± 1.95	≤0.001
CRF	9.33 ± 1.94	10.05 ± 2.6	≤0.001

**Table 2:** Bivariate correlations between ΔCH and each of age, sex, refraction, and preoperative CH.

Variable 1	Variable 2 (mmHg)	*r* ^†^	*p*
Age (yrs)	ΔCH	0.1	0.52
Sex	ΔCH	0.47	0.77
Refraction (D)	ΔCH	0.2	0.08
Pre-op CH (mmHg)	ΔCH	0.32	0.03

^†^Pearson's correlation coefficient.

**Table 3:** Bivariate correlations between ΔCRF and each of age, sex, refraction, and preoperative CRF.

Variable 1	Variable 2 (mmHg)	*r* ^†^	*p*
Age (yrs)	ΔCRF	0.26	0.09
Sex	ΔCRF	0.17	0.26
Refraction (D)	ΔCRF	−0.04	0.77
Pre-op CRF (mmHg)	ΔCRF	0.19	0.21

^†^Pearson's correlation coefficient.

**Table 4:** The mean differences in measured parameters for single muscle surgery versus 2-3 muscle surgeries.

Parameter (mmHg)	Single muscle (*N*=18)^†^	2-3 muscles (*N*=24)	*p*
ΔCH	−1.28 ± 1.5	−0.4 ± 1.49	0.07
ΔCRF	−0.91 ± 1.81	−0.58 ± 2.4	0.3

^†^Number of the eyes.

**Table 5:** The mean differences in measured parameters for weakening muscle surgery versus combined muscle surgery.

Parameter (mmHg)	Weakening group (*N*=23)^†^	Combined group (*N*=18)	*p*
ΔCH	−1.24 ± 1.77	−0.26 ± 1.07	0.04
ΔCRF	−0.93 ± 2.1	−0.5 ± 2.29	0.53

^†^Number of the eyes.

## Data Availability

The data used to support the findings of this study are available from the corresponding author upon request.
